# SIRT1 Expression in Human Gastrointestinal Tumors and Its Clinical Significance

**DOI:** 10.1002/cam4.71217

**Published:** 2025-09-29

**Authors:** Wenxuan Liu, Xinyi Li, Wenhong Deng

**Affiliations:** ^1^ Laboratory of General Surgery Renmin Hospital of Wuhan University Wuhan Hubei China; ^2^ Department of General Surgery The Second Affiliated Hospital of Anhui Medical University Hefei China

**Keywords:** clinical significance, colon cancer, gastric cancer, immunity, rectal cancer, SIRT1

## Abstract

**Objective:**

This study aimed to investigate the expression levels of SIRT1 protein in gastric (GC), colon (CC), and rectal cancer (RC) tissues and patient plasma, analyze its correlation with clinicopathological features and prognosis, and preliminarily explore its relationship with the tumor immune microenvironment.

**Methods:**

Plasma samples were collected from 198 gastrointestinal cancer patients (66 each of GC, CC, and RC) and 66 healthy volunteers. Additionally, cancerous and adjacent normal tissues were obtained from 45 of these patients (15 pairs for each cancer type). SIRT1 concentration in plasma was detected using Enzyme‐Linked Immunosorbent Assay (ELISA). SIRT1 protein expression in tissues was examined using Immunohistochemistry (IHC) and Western Blotting. Bioinformatic analysis was performed using TCGA and GEPIA databases. The correlation between SIRT1 and immune cell infiltration was analyzed via the TIMER database. Statistical analyses were conducted using SPSS software.

**Results:**

Plasma SIRT1 concentration was significantly higher in the GC group compared to healthy controls (*p* = 0.045), while it was significantly lower in the CC and RC groups (*p* = 0.007 and *p* = 0.009, respectively). In tissues, SIRT1 expression was up‐regulated in GC but down‐regulated in colorectal cancer (CC and RC) tissues (*p* < 0.05). SIRT1 expression levels were significantly correlated with clinicopathological features including tumor differentiation degree, depth of invasion, TNM stage, distant metastasis (in colorectal cancer), and lymph node metastasis (in RC) (*p* < 0.05). Survival analysis revealed that high SIRT1 expression was associated with poorer overall survival (OS) in GC patients (*p* = 0.032), while low expression was associated with poorer OS in RC patients (*p* = 0.027). Plasma SIRT1 levels showed significant correlations with various tumor markers (e.g., CEA, CA199, CA125, CA724). Furthermore, SIRT1 expression was positively correlated with the infiltration levels of immune cells such as CD4+ T cells, CD8+ T cells, macrophages, and neutrophils.

**Conclusion:**

SIRT1 expression in gastrointestinal tumors is tissue‐specific (upregulated in GC, downregulated in colorectal cancer). Its expression level is closely associated with malignant progression and patient prognosis, and it may be involved in the modulation of the tumor immune microenvironment. SIRT1 shows promise as a potential diagnostic biomarker and therapeutic target for gastrointestinal cancers.

## 1 Introduction

Gastrointestinal tumors, encompassing gastric, colon, and rectal cancers, represent some of the most prevalent malignant neoplasms of the digestive system [1]. Globally, there has been a notable increase in both the incidence and mortality rates of gastric and colorectal cancers, reflecting the substantial burden these diseases impose on public health. Colorectal cancer has the third greatest occurrence rate and the second highest fatality rate, whereas gastric cancer has the fifth highest occurrence rate, ranking behind colorectal cancer, and the fifth highest fatality rate, as well as the occurrence rate [2]. These figures demonstrate that these tumors pose a substantial risk to human health. Due to the insidious onset and unclear pathogenesis of gastrointestinal tumors, there are no obvious symptoms in the early stages. As a result, most symptoms manifest during the intermediate and advanced stages, leading to an unfavorable prognosis.

The gastrointestinal tumorigenesis and pathogenesis are intricate processes, governed by a plethora of distinct genes. Several researchers have concentrated on genes and their mechanisms linked to the development of gastrointestinal tumors in order to uncover potential medicines that target specific genes and can be tailored to individuals [3, 4]. Prior research has utilized various approaches to find promising biomarkers for the timely detection and prediction of gastrointestinal tumors [5]. However, the rationale behind this approach remains unclear, necessitating further investigation.

SIRT1 has been shown to be a type III histone deacetylase (HDAC) that is dependent on nicotinamide adenine dinucleotide (NAD+) for its functioning, as revealed by recent research [6]. A body of evidence indicates that protein deacetylation is a pivotal factor in gene expression and regulation, particularly in processes such as cell survival, senescence, apoptosis, differentiation, and other metabolic processes [7, 8]. A number of studies have demonstrated that SIRT1 expression is elevated in prostate, skin, squamous cell, and pancreatic carcinoma cancers [9, 10, 11, 12]. SIRT1 plays a role in the development of several diseases through its deacetylase activity [13, 14].

To gain further insight into the presence of SIRT1 in gastrointestinal tumors, this study examined the distribution of SIRT1 in gastric cancer (GC), colon cancer (CC), and rectal cancer (RC) tissues and normal tissues, as well as in plasma of patients with gastric, colon, and rectal cancers and healthy populations. This was achieved through the use of immunohistochemistry and ELISA analyses, which enabled the analysis of SIRT1 expression levels and the association of clinicopathological factors. The findings may offer novel insights and avenues for further investigation into the pathology of gastrointestinal tumors.

## 2 Materials and Methods

### 2.1 Collection and Storage of Plasma and Tissue Samples

From September 2021 to May 2024, plasma samples were collected from 198 patients with gastrointestinal tumors (66 each of gastric, colon, and rectal cancers) who underwent surgery and 66 healthy volunteers. Additionally, specimens of cancerous tissues were obtained from 45 of these patients (15 pairs of each of gastric, colon, and rectal cancers), as well as normal tissues. The samples were collected from the People's Hospital of Wuhan University (Wuhan, China).

The blood samples were placed in vacuum blood collection tubes that contained ethylenediaminetetraacetic acid (EDTA) anticoagulant. The tubes were then refrigerated at a temperature of 4°C. Within 2 h of collection, all samples were centrifuged at 4°C and 1000 g for 15 min. Afterwards, the liquid above the sediment was moved to empty Eppendorf tubes and kept at a temperature of −80°C. All specimens consisted of recently harvested tumor tissue and paracancerous tissue, which refers to normal mucosal tissue located at least 5 cm away from the tumor edge. The samples were placed in EP tubes that had been treated with a solution of 0.1% diethyl pyrocarbonate (DEPC) in water.

The following criteria were used to determine eligibility for inclusion in the study: (1) All patients were diagnosed with gastric, colon, or rectal cancer following histopathological examination and had no previous gastrointestinal surgery. (2) They received a complete laboratory examination and had complete clinical and pathological data. The following criteria must be met in order to be eligible for inclusion in the study: (1) Other malignant tumors or serious diseases of the gastrointestinal tract; (2) neoadjuvant chemotherapy in the last 3 months.

### 2.2 Enzyme‐Linked Immunosorbent Assay (ELISA)

Prior to analysis, all reagents, working standards, and samples were kept at room temperature for at least 30 min. Firstly, the samples and standards of different concentrations were added to the corresponding wells at 100 μL per well, respectively, and 100 μL of universal diluent was added to the blank wells. Incubate for 60 min at 37°C. Next, the solution containing the antibody that has been modified with biotin is introduced, and the process of incubation is prolonged for a duration of 60 min. Afterwards, the plate was cleansed, with each well undergoing a 60‐min period of incubation with a wash water buffer. The liquid was fully extracted at each stage. The plate was washed an additional five times following the previously specified technique. Afterward, 90 μL of substrate (TMB) was introduced into each well. The plate was then covered with a membrane, and the contents were left to incubate for 15 min in the absence of light. Before adding 50 μL of termination solution to each well, promptly evaluate the optical density value of each well at 450 nm.

### 2.3 Immunohistochemistry

Tumor and paracancerous tissues were collected from GC, CC, and RC patients in Renmin Hospital of Wuhan University, after we excluded samples without obvious tumor tissue. Fifteen pairs of each tumor type were collected to verify the differential expression of SIRT1. The expression of SIRT1 was assessed by immunohistochemistry (IHC). To enhance antigen retrieval, paraffin‐embedded tissue sections were deparaffinized and treated with citrate buffer at 100°C for 15 min. Following an overnight incubation at 4°C with the primary antibody (Proteintech, 13161‐1‐AP1:1000), the sections were individually and gently washed with a sufficient volume of PBS. Subsequently, they were treated with the suitable secondary antibody for 30 min at 37°C. The sections were stained with hematoxylin and restained with DAB following a second rinse in PBS. Immunohistochemical sections were examined using an Olympus BX63 microscope to assess the localization and intensity of staining. All slides were measured and photographed blindly using a light microscope. When taking pictures, make sure that the exposure time, aperture, and exposure conditions are the same for each picture by using a camera with manual controls that does not move any parts except for adjusting the focus and field of view. The AOD (integrated optical density/area) of the immunostained sections was measured quantitatively using the Image‐Pro Plus 6.0 software (Media Cybernetics Inc., Bethesda, USA). The median value was used to determine whether a group had high or low expression.

### 2.4 Western Blotting

Total cellular proteins were extracted using RIPA lysis buffer (G2002, Servicebio, Wuhan, China) and the obtained proteins were quantified using the BCA method (G2026, Servicebio, Wuhan, China). Individual proteins were isolated from the mixture using SDS‐PAGE. The isolated proteins were then transferred onto nitrocellulose (NC) membranes (Millipore, Massachusetts, USA). The membranes were then treated with primary antibodies specific for SIRT1 (13161‐1‐AP, Proteintech) and GAPDH (60004‐1‐Ig, Proteintech) for 16 h at 4°C. The membranes were then washed with TBST to remove the proteins from the membrane. The membranes were then washed with TBST and incubated with fluorescent secondary antibodies for 1 h at room temperature. After washing the membranes with TBST, the membranes were scanned with an Odyssey instrument and analyzed to calculate the relative expression of each index.

### 2.5 Statistical Methods

The data were analyzed utilizing the SPSS software (IBM SPSS Statistics, V21.0) and presented as the mean ± standard deviation. A paired samples *t*‐test was used to examine the expression of SIRT1 in gastrointestinal tumors and normal tissues, as well as the levels of SIRT1 in the plasma of gastrointestinal tumors and healthy controls. To evaluate the existence of variations in SIRT1 expression in tissues associated with a variety of clinical and pathological variables, a one‐way ANOVA was implemented. The correlation of SIRT1 levels with tumor markers was analyzed by Pearson's test. In order to determine whether or not there was a statistically significant difference, a *p*‐value of less than 0.05 was necessary.

## 3 Results

### 3.1 A Comparison of the General Conditions of the Gastrointestinal Tumor Group and the Normal Control Group

A cohort of 198 individuals diagnosed with gastrointestinal tumors and 66 healthy volunteers were enlisted to undergo plasma SIRT1 testing. The plasma SIRT1 expression was (4.96 ± 2.59) ng/mL in 66 patients with gastric cancer, 43 of whom were male and 23 of whom were female, and the age range was 34–76 (58.91 ± 9.78) years. Sixty‐six patients with colon cancer, aged 27–80 (57.96 ± 13.79) years, with plasma SIRT1 expression of (3.29 ± 1.43) ng/mL, consist of 39 males and 27 females. Plasma SIRT1 expression was 3.33 ± 1.35 ng/mL in 66 patients with rectal cancer, including 36 males and 30 females, aged 22–83 (59.50 ± 15.40) years. The healthy control group consisted of 34 females and 32 males, with ages ranging from 34 to 74 years old (mean age: 54.52 ± 9.65). The plasma SIRT1 expression was (4.14 ± 4.14) ng/mL, as indicated in Table S1.

**FIGURE 1:**
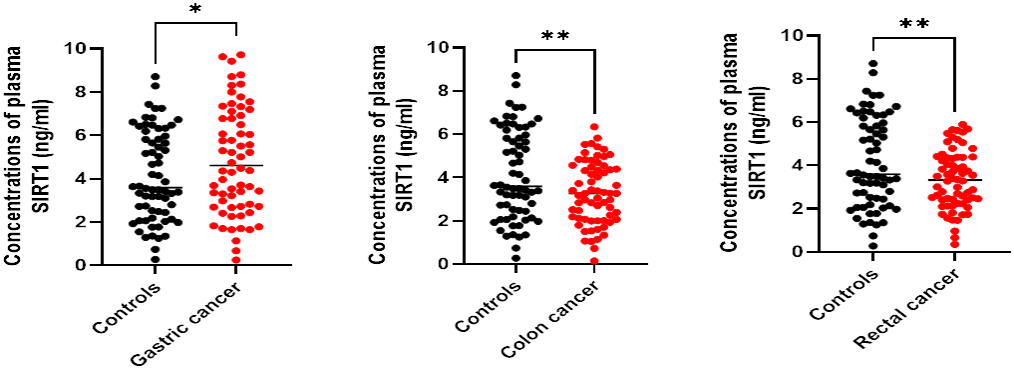
SIRT1 levels in plasma of GC, CC, and RC patients, ELISA analysis showed that serum SIRT1 levels were significantly higher in GC patients than in healthy controls and lower in CC and RC patients than in the healthy group.

The plasma concentration of SIRT1 was significantly higher in the GC group [(4.96 ± 2.59) ng/mL vs. (4.14 ± 2.08) ng/mL, *p* = 0.045] compared to the normal control group. Compared to the normal control group, the CC group's plasma concentration of SIRT1 was significantly lower [(3.29 ± 1.43) ng/mL vs. (4.14 ± 2.08) ng/mL, *p* = 0.007]. In the RC group, the plasma concentration of SIRT1 was significantly lower than that of the normal control group [(3.33 ± 1.35) ng/mL vs. (4.14 ± 2.08) ng/mL, *p* = 0.009]. This disparity was statistically significant (Figure 1).

### 3.2 Correlation Between SIRTI Levels in Plasma and Clinicopathological Parameters in Gastrointestinal Tumors

There was a statistically significant difference in the plasma levels of SIRT1 between male and female GC patients [(5.16 ± 2.58) ng/mL vs. (2.58 ± 2.64) ng/mL, *p* = 0.41]. When comparing the groups with medium and high degrees of differentiation, the plasma concentration of SIRT1 in the low degree of differentiation group was significantly higher [(6.16 ± 2.47) ng/mL vs. (3.53 ± 1.95) ng/mL, *p* < 0.01]. The plasma SIRT1 concentration was found to be lower in patients with infiltration depths of mucosa and muscular layer than in those with infiltration depths of the entire layer. The mean concentration was (4.28 ± 2.53) ng/mL in the former group and (5.74 ± 2.47) ng/mL in the latter, with a statistically significant variation (*p* = 0.021). The plasma SIRT1 concentration in the group with metastatic TNM stage I + II was found to be lower than that in the group with stage III + IV [(4.29 ± 2.64) ng/mL vs. (5.59 ± 2.42) ng/mL, *p* = 0.025], indicating a difference that is statistically noteworthy. Among patients with gastric cancer (GC), there was no statistically significant variation in the plasma SIRT1 levels according to age, the presence of distant metastases from the tumor, or the presence of lymph node metastases. Moreover, there was no discernible relationship between the plasma SIRT1 levels and the existence of lymph node metastases (*p* > 0.05; Table 1).

**TABLE 1:** Association between SIRT1 expression and clinicopathological data of patients with gastric cancer.

Variable	Case	SIRT1 (ng/mL)	*p*
Age			
≤ 60 years	36	5.02 ± 2.68	0.847
> 60 years	30	4.90 ± 2.52	
Gender			
Male	43	5.16 ± 2.58	0.412
Female	23	2.58 ± 2.64	
Differentiation			
Low	36	6.16 ± 2.47	0.000
Medium + high	30	3.53 ± 1.95	
Depth of invasion			
Mucous membranes + muscle layer	35	4.28 ± 2.53	0.021
Whole layer	31	5.74 ± 2.47	
TNM stage			
I + II	32	4.29 ± 2.64	0.025
III + IV	34	5.59 ± 2.42	
Distant metastasis			
Yes	26	5.58 ± 2.58	0.120
No	40	4.56 ± 2.55	
Lymph node metastasis			
Yes	36	5.26 ± 2.64	0.308
No	30	4.61 ± 2.53	

The quantities of SIRT1 in the plasma of patients diagnosed with colon cancer exhibited a noteworthy distinction between the low and moderately differentiated groups, as well as the highly differentiated group [(2.48 ± 1.14) ng/mL vs. (3.69 ± 1.40) ng/mL, *p* < 0.01]. Plasma SIRT1 concentration was higher in the metastatic TNM staging I + II group than in the staging III + IV group [(3.80 ± 1.41) ng/mL vs. (2.87 ± 1.33) ng/mL, *p* = 0.008]. The group with distant tumor metastasis was lower than the group without distant metastasis [(2.78 ± 1.42) ng/mL vs. (3.69 ± 1.33) ng/mL, *p* = 0.01]. Age, sex, degree of infiltration, and the presence or absence of lymph node metastases in CC patients did not significantly correlate with plasma SIRT1 levels (*p* > 0.05; Table 2).

**TABLE 2:** Association between SIRT1 expression and clinicopathological data of patients with colon cancer.

Variable	Case	SIRT1 (ng/mL)	*p*
Age			
≤ 60 years	38	3.23 ± 1.46	0.710
> 60 years	28	3.37 ± 1.42	
Gender			
Male	39	3.33 ± 1.40	0.781
Female	27	3.23 ± 1.51	
Differentiation			
Low	22	2.48 ± 1.14	0.001
Medium + High	44	3.69 ± 1.40	
Depth of invasion			
Mucous membranes + muscle layer	38	3.51 ± 1.54	0.148
Whole layer	28	2.99 ± 1.24	
TNM stage			
I + II	30	3.80 ± 1.41	0.008
III + IV	36	2.87 ± 1.33	
Distant metastasis			
Yes	29	2.78 ± 1.42	0.010
No	37	3.70 ± 1.33	
Lymph node metastasis			
Yes	31	3.53 ± 1.44	0.203
No	35	3.08 ± 1.42	

SIRT1 plasma concentration in rectal cancer patients was found to be lower in the low differentiation group [(2.84 ± 1.35) ng/mL vs. (3.69 ± 1.25) ng/mL, *p* = 0.011] than in the moderate and high differentiation groups. Plasma SIRT1 concentration was higher in the group with metastatic TNM staging I + II than in the group with staging III + IV [(3.64 ± 1.42) ng/mL vs. (2.94 ± 1.17) ng/mL, *p* = 0.036]. The plasma concentrations of SIRT1 were shown to be lower in individuals with lymph node metastases compared to those without lymph node metastases [(2.67 ± 1.36) ng/mL vs. (3.74 ± 1.19) ng/mL, *p* = 0.001]. Plasma SIRT1 concentrations in RC patients did not show any meaningful connection with age, sex, degree of infiltration, or presence of distant metastases (*p* > 005; Table 3).

**TABLE 3:** Association between SIRT1 expression and clinicopathological data of patients with rectal cancer.

Varible	Case	SIRT1 (ng/mL)	*p*
Age			
≤ 60 years	31	3.34 ± 1.35	0.957
> 60 years	35	3.32 ± 1.38	
Gender			
Male	36	3.02 ± 1.13	0.075
Female	30	3.61 ± 1.49	
Differentiation			
Low	28	2.84 ± 1.35	0.011
Medium + high	38	3.69 ± 1.25	
Depth of invasion			
Mucous membranes + muscle layer	39	3.41 ± 1.35	0.569
Whole layer	27	3.22 ± 1.38	
TNM stage			
I + II	37	3.64 ± 1.42	0.036
III + IV	29	2.94 ± 1.17	
Distant metastasis			
Yes	27	3.06 ± 1.28	0.180
No	39	3.52 ± 1.39	
Lymph node metastasis			
Yes	25	2.67 ± 1.36	0.001
No	41	3.74 ± 1.19	

### 3.3 SIRT1 Expression in Human Gastrointestinal Tumors and Normal Tissues

**FIGURE 2:**
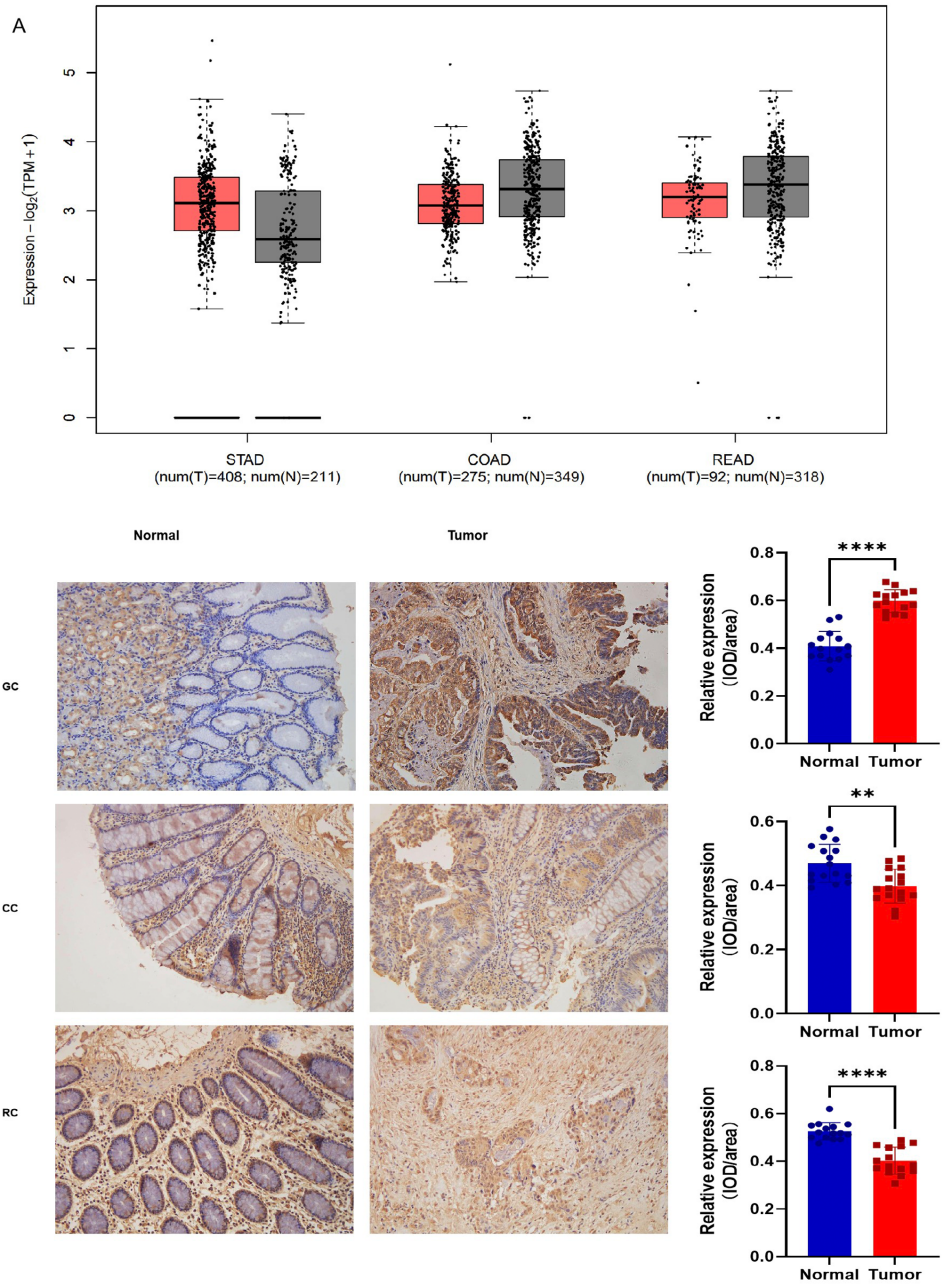
SIRT1 expression in human GC, CC, and RC patients and normal tissues. (A) Box plots comparing SIRT1 expression in GC, CC, and RC patients from TCGA database. (B) Immunohistochemical analyses of tumor tissues and normal tissues of GC, CC, and RC patients. (C) Immunohistochemical statistical plots.

We performed a comprehensive investigation of the levels of manifestation of SIRT1 in malignant tissues and normal samples obtained from the TCGA STAD, COAD, and READ datasets utilizing the GEPIA database. According to the findings, SIRT1 was shown to be increased in gastric cancer patients and decreased in colon and rectal cancer patients compared to normal samples, as depicted in Figure 2A. Immunohistochemical staining was employed to identify the presence of SIRT1 in gastric cancer (GC), colon cancer (CC), rectal cancer (RC) tissues, as well as normal tissues. The protein SIRT1 was mostly found in the nucleus, and its expression was considerably higher in gastric cancer cells compared to normal tissues (*p* < 0.0001). When compared to the surrounding tissues, the expression of SIRT1 in CC and RC tissues was significantly reduced (*p* = 0.002, *p* < 0.0001), as seen in Figure 2B,C. This finding represents a significant departure from the surrounding tissues. Western blotting (WB) showed that the protein level of SIRT1 was significantly upregulated in samples from patients with GC, whereas it was downregulated in patients with colorectal cancer (Figure S1).

### 3.4 Relationship Between SIRT1 and Prognosis of Gastrointestinal Tumors

**FIGURE 3:**
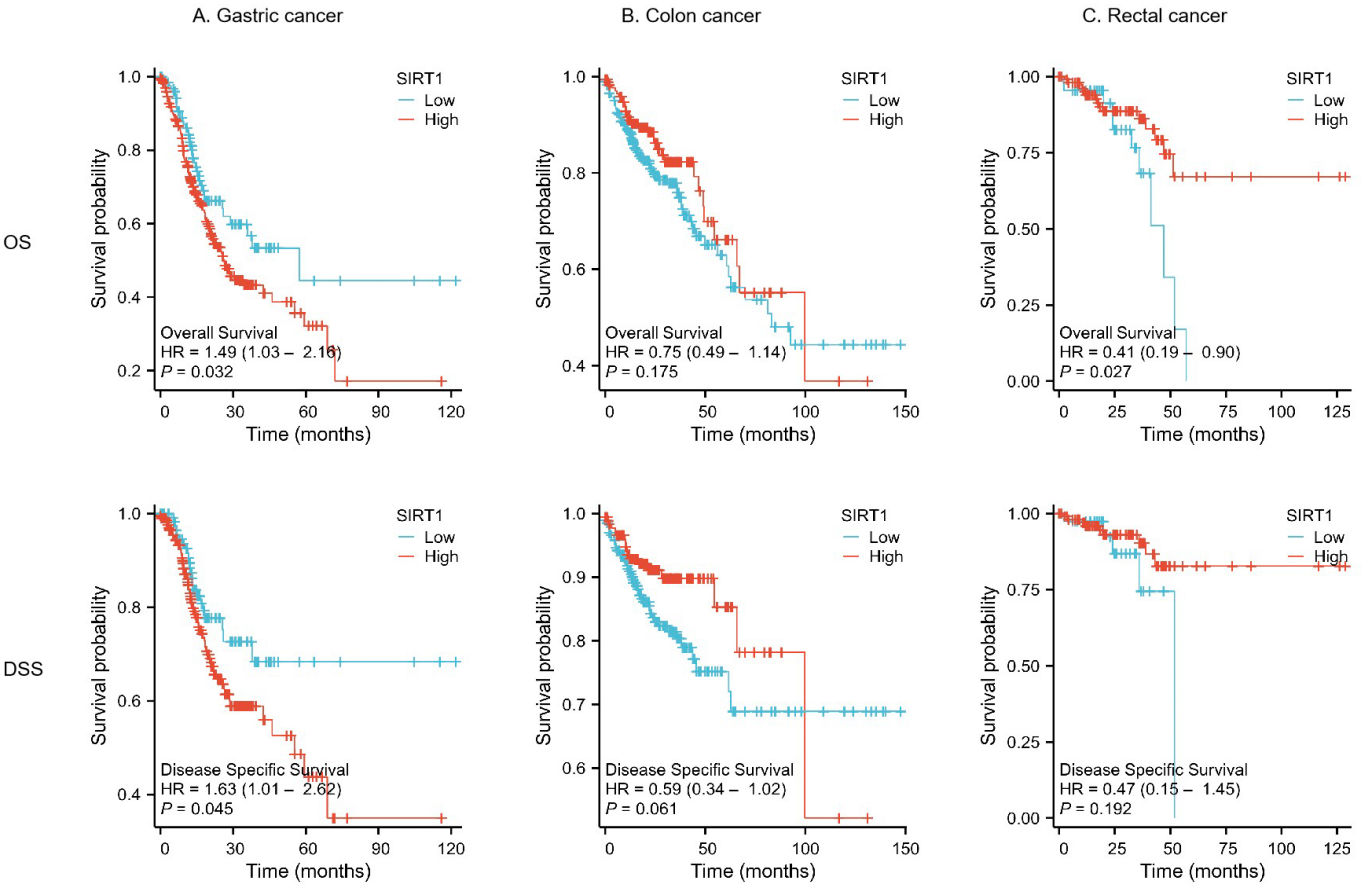
Relationship between SIRT1 expression and survival. (A) Relationship between SIRT1 and OS, DSS in GC patients. (B) Relationship between SIRT1 and OS, DSS in CC patients. (C) Relationship between SIRT1 and OS, DSS in RC patients.

It was determined whether or not there was a connection between the levels of SIRT1 expression and the prognosis of patients who were diagnosed with gastrointestinal malignancies via the course of an inquiry. It was found that in gastric cancer, higher SIRT1 expression levels were strongly linked to unfavorable overall survival (OS) in GC (*p* = 0.032, HR = 1.49) (Figure 3A). In rectal cancer, lower SIRT1 expression levels were observed to be significantly associated with unfavorable overall survival (OS) in gastric cancer (*p* = 0.027, HR = 0.41) (Figures 3B and 3C). Nevertheless, there was no noticeable disparity observed in the occurrence of colon cancer. Furthermore, lower SIRT1 expression levels were observed to be significantly correlated with disease‐specific survival (DSS) in gastric cancer (*p* = 0.045, HR = 0.63), but there was no notable disparity detected in cases of colon and rectal malignancies. These data indicate that increased expression of SIRT1 may be a predictor of worse overall survival (OS) in patients with gastric cancer, whereas reduced SIRT1 may be a predictor of worse OS in patients with rectal cancer (Figure 3).

### 3.5 Correlation Between Plasma SIRT1 Levels and Tumor‐Related Markers in Patients With Gastrointestinal Tumors

The data presented in Table 4 demonstrates a direct correlation between the plasma SIRT1 level in patients with gastric cancer and the commonly used gastrointestinal tumor markers CEA (*r* = 0.255, *p* = 0.039), CA125 (*r* = 0.260, *p* = 0.035), and CA724 (*r* = 0.268, *p* = 0.030). Nevertheless, there is no substantial association seen between the plasma SIRT1 level and CA199, SCC, AFP, AFU, ALT, and AST (*p* > 0.05). Additionally, there is no significant correlation between the plasma SIRT1 level and CEA (*r* = −0.251, *p* = 0.042). Table 5 shows that there was no statistically significant connection (*p* > 0.05) observed with CA199, SCC, AFP, AFU, ALT, and AST. The plasma level of SIRT1 in colon cancer patients showed a negative association with CEA (*r* = −0.251, *p* = 0.042) and CA199 (*r* = −0.342, *p* = 0.005). However, Table 4 demonstrates that there was no noteworthy link detected between CA125, CA724, SCC, AFP, AFU, ALT, and AST (*p* > 0.05). Table 5 indicates that there was no statistically significant correlation (*p* > 0.05) identified between CA125, CA724, SCC, AFP, AFU, ALT, and AST. The plasma SIRT1 level of rectal cancer patients was negatively correlated with CEA (*r* = −0.250, *p* = 0.043), CA199 (*r* = −0.365, *p* = 0.003); the variables CA125, CA724, SCC, AFP, AFU, ALT, and AST did not exhibit a statistically significant connection (*p* > 0.05) with each other, as indicated in Table 6.

**TABLE 4:** Correlation between serum SIRT1 and tumor markers in patients with gastric cancer.

Variable	*r*	*p*
CEA	0.255	0.039
CA125	0.260	0.035
CA199	0.177	0.155
CA724	0.268	0.030
SCC	0.210	0.091
AFP	−0.036	0.775
AFU	−0.149	0.233
ALT	0.034	0.786
AST	−0.140	0.261

**TABLE 5:** Correlation between serum SIRT1 and tumor markers in patients with colon cancer.

Variable	*r*	*p*
CEA	−0.251	0.042
CA125	−0.168	0.179
CA199	−0.342	0.005
CA724	−0.183	0.140
SCC	0.104	0.406
AFP	0.107	0.395
AFU	0.208	0.094
ALT	−0.125	0.318
AST	0.056	0.654

**TABLE 6:** Correlation between serum SIRT1 and tumor markers in patients with rectal cancer.

Variable	*r*	*p*
CEA	−0.250	0.043
CA125	−0.068	0.588
CA199	−0.365	0.003
CA724	−0.044	0.728
SCC	0.023	0.855
AFP	−0.153	0.220
AFU	0.115	0.359
ALT	−0.014	0.913
AST	0.008	0.949

### 3.6 SIRT1 in Gastrointestinal Tumor‐Infiltrating Immune Cells

**FIGURE 4:**
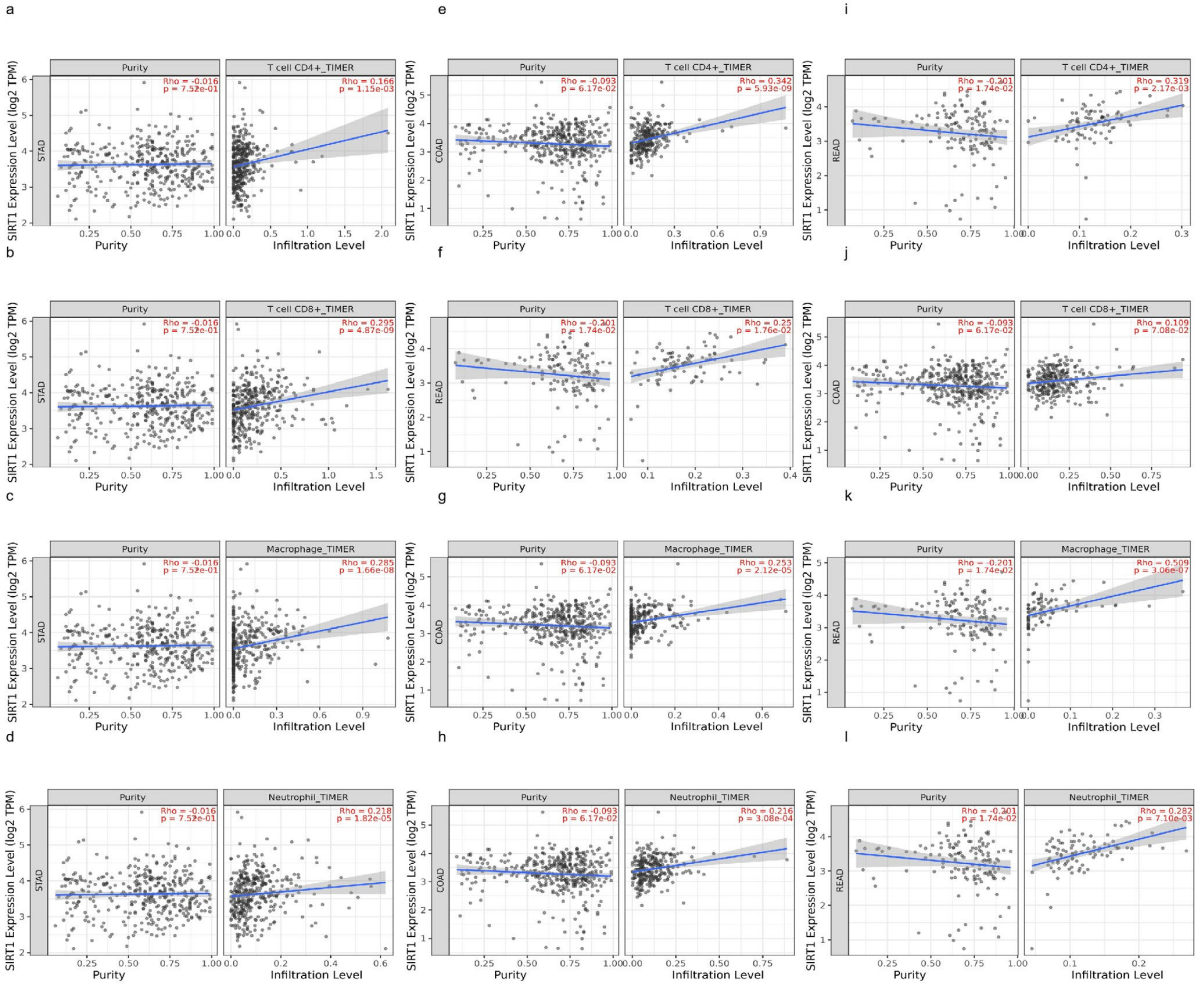
SIRT1 in tumor‐infiltrating immune cells. (a) Relationship between SIRT1 and gastric cancer CD4+ T cells. (b) Relationship between SIRT1 and gastric cancer CD8+ T cells. (c) Relationship between SIRT1 and gastric cancer macrophages. (d) Relationship between SIRT1 and gastric cancer neutrophils. (e) Relationship between SIRT1 and colon cancer CD4+ T cells. (f) Relationship between SIRT1 and colon cancer CD8+ T cells. (g) Relationship between SIRT1 and colon cancer macrophages. (h) Relationship between SIRT1 and colon cancer neutrophils. (i) Relationship between SIRT1 and rectal cancer CD4+ T cells. (j) Relationship between SIRT1 and rectal cancer CD8+ T cells. (k) Relationship between SIRT1 and rectal cancer macrophages. (l) Relationship between SIRT1 and rectal cancer neutrophils.

Through the use of the TIMER database, we investigated the link between this gene and immune cell infiltration in order to discover the connection that exists between SIRT1 and the immunomodulation of gastrointestinal tumors. In cases of gastric cancer, there was a positive correlation between SIRT1 and CD4+ T cells (*r* = 0.166), CD8+ T cells (*r* = 0.295), macrophages (*r* = 0.285), and neutrophils (*r* = 0.218). Colon cancer showed a positive correlation between SIRT1 and CD4+ T cells (*r* = 0.342), CD8+ T cells (*r* = 0.25), macrophages (*r* = 0.253), and neutrophils (*r* = 0.216), which is promising. Rectal cancer patients showed a positive correlation between SIRT1 and CD4+ T cells (*r* = 0.319), CD8+ T cells (*r* = 0.109), macrophages (*r* = 0.509), and neutrophils (*r* = 0.282) (Figure 4).

## 4 Discussion

In our recent study, we investigated the high and low expression of SIRT1, a NAD+‐dependent histone deacetylase, also known as HDAC class III enzyme [6], in GC, CC, and RC cancers and its association with tumor biological characteristics. In our study, we found that the expression level of SIRT1 was higher in gastric cancer patients and lower in colon and rectal cancer patients. In gastric cancer tissues, high expression of SIRT1 was closely associated with the degree of tumor differentiation, depth of invasion, and TNM stage (*p* < 0.05). In contrast, in colon and rectal cancer tissues, low expression of SIRT1 was closely associated with tumor differentiation, TNM stage, and distant metastasis (*p* < 0.05). In addition, the expression level of SIRT1 was significantly correlated with tumor markers such as CA724, CEA, and CA125 in gastric cancer patients, and CEA and CA199 in colon and rectal cancer patients (*p* < 0.05), suggesting that SIRT1 may not only be a new target for cancer therapy, but also an important tool for clinical use in cancer diagnosis.

SIRT1 plays an important role in the regulation of cellular senescence, energy metabolism, and tumor growth [15]. It slows down the process of cellular senescence by enhancing FOXO‐induced cell cycle arrest and stress resistance through deacetylation, while attenuating FOXO‐induced apoptosis [7, 16]. The histidine residue at position 363 in the SIRT1 protein is essential for its deacetylation activity, and this activity is regulated by the ratio of NAD+ to NADH in the cytoplasm [17]. SIRT1 is also involved in tumor growth by deacetylating the lysine at position 382 of the p53 protein, which reduces its transcriptional activity and p53‐dependent apoptosis induction [18]. Although previous studies have shown high expression of SIRT1 in colorectal cancer [19], the results of our clinical samples showed low expression of SIRT1 in colorectal cancer, a finding that was further validated by immunohistochemistry and Western blotting.

In this study, we conducted an extensive investigation into the role of SIRT1 in gastrointestinal cancers through immunohistochemistry and ELISA, alongside data from the TCGA and TIMER databases. IHC, performed on surgically resected tumor specimens, provides critical insights into tumor biology, including the degree of cellular differentiation, invasiveness, and potential prognostic markers. For example, the overexpression status of HER2 (human epidermal growth factor receptor 2) is an important predictor in the treatment of gastric cancer, and detecting the expression level of HER2 protein by IHC can guide clinicians in deciding whether or not to use targeted therapy against HER2 [20]. In addition, IHC can be used to detect immune cell infiltration in the tumor microenvironment, which is important for assessing the efficacy of immunotherapy and predicting patient prognosis [21]. Our results suggest that SIRT1 is importantly associated with certain clinical stages and differentiation status, as other studies have shown that SIRT1 expression is higher in the early stages of tumors [22], whereas our results focused on the advanced stages, which may suggest a dual role for SIRT1 at different stages of cancer development.

The development of gastrointestinal tumors is strongly influenced by dietary habits, lifestyle factors, and geographical location [23]. In this study, we observed a marked upregulation of SIRT1 expression in gastric cancer tissues, while a significant downregulation was evident in colon and rectal cancers compared with normal mucosal tissues. Although gender itself did not significantly affect SIRT1 expression, gastric cancer patients showed higher levels in men than in women, which may be related to the higher incidence of gastrointestinal tumors in males [24]. Moreover, indicators of cancer progression such as tumor differentiation, depth of infiltration, and lymph node metastasis were closely associated with SIRT1 expression [25]. Overexpression of SIRT1 in gastric cancer tissues was found to have a significant correlation with tumor differentiation, infiltration depth, and TNM stage (*p* < 0.05). Specifically, overexpression of SIRT1 in gastric cancer correlated with tumor differentiation, infiltration depth, and TNM stage (*p* < 0.05). In contrast, low SIRT1 expression in colon cancer was significantly related to differentiation, TNM stage, and distant metastasis (*p* < 0.05), while in rectal cancer, reduced SIRT1 expression was associated not only with differentiation and TNM stage but also with lymph node metastasis (*p* < 0.05). These findings suggest that SIRT1 may play a key role in the infiltration and spread of gastrointestinal tumors [26].

Furthermore, elevated SIRT1 expression in gastric cancer showed a strong correlation with both OS and DSS (*p* < 0.05), providing compelling evidence that SIRT1 is a gene associated with gastric cancer progression. Tumor‐associated markers such as CA724, CA199, CEA, and CA125 also serve as important indicators of tumor presence and progression [27]. It was revealed that there were significant relationships between the levels of SIRT1 and CA724, CEA, and CA125 in patients with gastric cancer (*p* < 0.05). Similarly, significant correlations were found between SIRT1 levels and CEA, CA199 in both colon and rectal cancer patients (*p* < 0.05). In the framework of the tumor microenvironment (TME), tumor‐infiltrating immune cells (TICs) have a high correlation with clinical outcomes and could either control tumor development in one direction or another [28]. With regard to malignancies of the stomach, colon, and rectal regions, SIRT1 was shown to have a positive correlation with CD4+ T cells, CD8+ T cells, macrophages, and neutrophils in this particular study. The observations indicate that SIRT1 has a substantial role in regulating the biological activity of gastrointestinal cancer cells [29].

This also confirms why the expression of SIRT1 was relatively low in colorectal cancer in our study. ELISA protocols are easy to perform, take very little time to perform, and have high sensitivity and specificity for monitoring changes in the levels of tumor markers such as CEA (carcinoembryonic antigen) and CA19‐9 [30], which may be associated with tumor recurrence, metastasis, or response to treatment.

While previous research has extensively explored the role of SIRT1 in gastric and colorectal cancers, our study offers novel insights by directly comparing SIRT1 expression across three major gastrointestinal cancers: gastric, colon, and rectal cancers. This comparative analysis is important as it provides a comprehensive understanding of how SIRT1 functions differently across various gastrointestinal tumor types. Moreover, our study goes beyond the simple characterization of SIRT1 expression levels, integrating multiple experimental techniques (immunohistochemistry, ELISA, Western blotting) and bioinformatic data from TCGA and TIMER to provide a robust and multi‐dimensional view of SIRT1's role. We also focus on the clinical implications of SIRT1 expression, demonstrating its potential as both a prognostic biomarker and therapeutic target in gastrointestinal cancers. Furthermore, by investigating the link between SIRT1 expression and immune cell infiltration in the tumor microenvironment, our study offers new insights into the immunological implications of SIRT1, an aspect that has not been fully explored in previous research. Overall, our findings contribute to a deeper understanding of SIRT1's role in gastrointestinal tumor progression and its potential as a target for future clinical interventions.

However, our study faces inherent limitations. The clinical sample size is insufficient, and there is a scarcity of in vitro and in vivo experiments to substantiate our findings. The relationship between SIRT1 expression and cancer progression, as well as its underlying mechanisms, remains elusive. Moving forward, we aim to expand our clinical sample cohort to validate our clinical observations and explore potential mechanisms. Further investigation is warranted to establish the link between SIRT1 and cancer progression, facilitating the development of novel strategies for early detection and targeted treatment of gastrointestinal cancers.

## Author Contributions

**Wenxuan Liu:** methodology, data curation, writing – original draft, software. **Xinyi Li:** writing – original draft, data curation. **Wenhong Deng:** writing – review and editing.

## Ethics Statement

Research has been performed in accordance with the Declaration of Helsinki. All methods were performed in accordance with the relevant guidelines and regulations. All patients provided their informed consent in writing preoperatively. Ethical approval for this study was obtained from the Clinical Research Ethics Committee of Wuhan University People's Hospital (No. WDRY2022‐K258).

## Consent

Gastric, colorectal, and rectal cancer samples and normal tissues were obtained from Wuhan University People's Hospital.

## Conflicts of Interest

The authors declare no conflicts of interest.

## Supporting information


**Figure S1:** Expression levels of SIRT1 in gastric cancer, colon cancer, and rectal cancer. Compared to normal tissue, SIRT1 expression was higher in gastric cancer patients and lower in colon and rectal cancer patients.
**Table S1:**. Baseline characteristics of the study subject.

## Data Availability

All of the data we used in this study were publicly available as described in the Section 2.

## References

[1] K. D. Miller , L. Nogueira , T. Devasia , et al., “Cancer Treatment and Survivorship Statistics, 2022,” CA: A Cancer Journal for Clinicians 72 (2022): 409–436, 10.3322/caac.21731. 35736631

[2] R. L. Siegel , K. D. Miller , and A. Jemal , “Cancer Statistics, 2020,” CA: A Cancer Journal for Clinicians 70 (2020): 7–30, 10.3322/caac.21590. 31912902

[3] T. Bou Kheir , E. Futoma‐Kazmierczak , A. Jacobsen , et al., “miR‐449 Inhibits Cell Proliferation and Is Down‐Regulated in Gastric Cancer,” Molecular Cancer 10 (2011): 29, 10.1186/1476-4598-10-29. 21418558 PMC3070685

[4] C. Travelli , F. M. Consonni , S. Sangaletti , et al., “Nicotinamide Phosphoribosyltransferase Acts as a Metabolic Gate for Mobilization of Myeloid‐Derived Suppressor Cells,” Cancer Research 79 (2019): 1938–1951, 10.1158/0008-5472.Can-18-1544. 30777853

[5] H. Lee , M. Song , N. Shin , et al., “Diagnostic significance of serum HMGB1 in colorectal carcinomas,” PLoS One 7 (2012): e34318, 10.1371/journal.pone.0034318. 22496788 PMC3319566

[6] M. Kaeberlein , M. McVey , and L. Guarente , “The SIR2/3/4 Complex and SIR2 Alone Promote Longevity in *Saccharomyces cerevisiae* by Two Different Mechanisms,” Genes & Development 13 (1999): 2570–2580, 10.1101/gad.13.19.2570. 10521401 PMC317077

[7] M. Fadri‐Moskwik , K. N. Weiderhold , A. Deeraksa , et al., “Aurora B Is Regulated by Acetylation/Deacetylation During Mitosis in Prostate Cancer Cells,” FASEB Journal 26 (2012): 4057–4067, 10.1096/fj.12-206656. 22751009 PMC3448774

[8] C. Feng , X. Jin , Y. Han , et al., “Expression and Prognostic Analyses of ITGA3, ITGA5, and ITGA6 in Head and Neck Squamous Cell Carcinoma,” Medical Science Monitor 26 (2020): e926800, 10.12659/msm.926800. 33099569 PMC7594586

[9] C. A. Bradbury , F. L. Khanim , R. Hayden , et al., “Histone Deacetylases in Acute Myeloid Leukaemia Show a Distinctive Pattern of Expression That Changes Selectively in Response to Deacetylase Inhibitors,” Leukemia 19 (2005): 1751–1759, 10.1038/sj.leu.2403910. 16121216

[10] Y. Hida , Y. Kubo , K. Murao , and S. Arase , “Strong Expression of a Longevity‐Related Protein, SIRT1, in Bowen's Disease,” Archives of Dermatological Research 299 (2007): 103–106, 10.1007/s00403-006-0725-6. 17180656

[11] H. Huang , S. Wang , H. Xia , et al., “Lactate Enhances NMNAT1 Lactylation to Sustain Nuclear NAD(+) Salvage Pathway and Promote Survival of Pancreatic Adenocarcinoma Cells Under Glucose‐Deprived Conditions,” Cancer Letters 588 (2024): 216806, 10.1016/j.canlet.2024.216806. 38467179

[12] X. W. Zhang , L. Li , M. Liao , et al., “Thermal Proteome Profiling Strategy Identifies CNPY3 as a Cellular Target of Gambogic Acid for Inducing Prostate Cancer Pyroptosis,” Journal of Medicinal Chemistry 67 (2024): 10005–10011, 10.1021/acs.jmedchem.4c00140. 38511243

[13] C. Philippe , M. Jaud , K. Féral , et al., “Pivotal Role of the Endoplasmic Reticulum Stress‐Related XBP1s/miR‐22/SIRT1 Axis in Acute Myeloid Leukemia Apoptosis and Response to Chemotherapy,” Leukemia 38 (2024): 1764–1776, 10.1038/s41375-024-02321-8. 38909090 PMC11286524

[14] I. M. M. van Leeuwen , M. Higgins , J. Campbell , et al., “Correction: Modulation of p53 C‐Terminal Acetylation by Mdm2, p14^ARF^, and Cytoplasmic SirT2,” Molecular Cancer Therapeutics 22 (2023): 1503, 10.1158/1535-7163.Mct-23-0759. 38037420

[15] Q. J. Wu , T. N. Zhang , H. H. Chen , et al., “The Sirtuin Family in Health and Disease,” Signal Transduction and Targeted Therapy 7 (2022): 402, 10.1038/s41392-022-01257-8. 36581622 PMC9797940

[16] C. Feng , W. Mao , C. Yuan , P. Dong , and Y. Liu , “Nicotine‐Induced CHRNA5 Activation Modulates CES1 Expression, Impacting Head and Neck Squamous Cell Carcinoma Recurrence and Metastasis via MEK/ERK Pathway,” Cell Death & Disease 15 (2024): 785, 10.1038/s41419-024-07178-4. 39472448 PMC11522702

[17] M. C. Motta , N. Divecha , M. Lemieux , et al., “Mammalian SIRT1 Represses Forkhead Transcription Factors,” Cell 116 (2004): 551–563, 10.1016/s0092-8674(04)00126-6. 14980222

[18] Y. Ma , H. Nie , H. Chen , et al., “NAD^+^/NADH Metabolism and NAD^+^‐Dependent Enzymes in Cell Death and Ischemic Brain Injury: Current Advances and Therapeutic Implications,” Current Medicinal Chemistry 22 (2015): 1239–1247, 10.2174/0929867322666150209154420. 25666794

[19] L. Kriegl , M. Vieth , T. Kirchner , and A. Menssen , “Up‐Regulation of c‐MYC and SIRT1 Expression Correlates With Malignant Transformation in the Serrated Route to Colorectal Cancer,” Oncotarget 3 (2012): 1182–1193, 10.18632/oncotarget.628. 23045412 PMC3717960

[20] J. Wang , Y. Liu , Q. Zhang , et al., “Disitamab Vedotin, a HER2‐Directed Antibody‐Drug Conjugate, in Patients With HER2‐Overexpression and HER2‐Low Advanced Breast Cancer: A Phase I/Ib Study,” Cancer Communications 44 (2024): 833–851, 10.1002/cac2.12577. 38940019 PMC11260767

[21] Y. Chen , K. Jia , X. Chong , et al., “Implications of PD‐L1 Expression on the Immune Microenvironment in HER2‐Positive Gastric Cancer,” Molecular Cancer 23 (2024): 169, 10.1186/s12943-024-02085-w. 39164705 PMC11334343

[22] T. W. Wang , E. Chern , C. W. Hsu , K. C. Tseng , and H. M. Chao , “SIRT1‐Mediated Expression of CD24 and Epigenetic Suppression of Novel Tumor Suppressor miR‐1185‐1 Increases Colorectal Cancer Stemness,” Cancer Research 80 (2020): 5257–5269, 10.1158/0008-5472.Can-19-3188. 33046442

[23] J. Boyer , S. Sedik , M. Egger , et al., “Performance of the Clarus Aspergillus Galactomannan Enzyme Immunoassay Prototype for the Diagnosis of Invasive Pulmonary Aspergillosis in Serum,” Mycoses 67 (2024): e13756, 10.1111/myc.13756. 38886163

[24] Y. Yao , L. Liu , G. Guo , Y. Zeng , and J. S. Ji , “Interaction of Sirtuin 1 (SIRT1) Candidate Longevity Gene and Particulate Matter (PM2.5) on All‐Cause Mortality: A Longitudinal Cohort Study in China,” Environmental Health 20 (2021): 25, 10.1186/s12940-021-00718-x. 33715628 PMC7958462

[25] X. Yao , G. Zhao , H. Yang , X. Hong , L. Bie , and G. Liu , “Overexpression of High‐Mobility Group Box 1 Correlates With Tumor Progression and Poor Prognosis in Human Colorectal Carcinoma,” Journal of Cancer Research and Clinical Oncology 136 (2010): 677–684, 10.1007/s00432-009-0706-1. 19898867 PMC11917171

[26] Y. Huang , M. Shao , X. Teng , et al., “Inhibition of CD38 Enzymatic Activity Enhances CAR‐T Cell Immune‐Therapeutic Efficacy by Repressing Glycolytic Metabolism,” Cell Reports Medicine 5 (2024): 101400, 10.1016/j.xcrm.2024.101400. 38307031 PMC10897548

[27] E. C. Smyth , M. Nilsson , H. I. Grabsch , N. C. van Grieken , and F. Lordick , “Gastric Cancer,” Lancet 396 (2020): 635–648, 10.1016/s0140-6736(20)31288-5. 32861308

[28] Q. L. Ou , Y. L. Chang , J. H. Liu , et al., “Mapping the Intellectual Structure and Landscape of Colorectal Cancer Immunotherapy: A Bibliometric Analysis,” Human Vaccines & Immunotherapeutics 20 (2024): 2323861, 10.1080/21645515.2024.2323861. 38497584 PMC10950274

[29] Y. Jiang , Y. Wang , G. Chen , et al., “Nicotinamide Metabolism Face‐Off Between Macrophages and Fibroblasts Manipulates the Microenvironment in Gastric Cancer,” Cell Metabolism 36 (2024): 1806–1822.e11, 10.1016/j.cmet.2024.05.013. 38897198

[30] B. L. Huang , L. F. Wei , Y. W. Lin , et al., “Serum IGFBP‐1 as a Promising Diagnostic and Prognostic Biomarker for Colorectal Cancer,” Scientific Reports 14 (2024): 1839, 10.1038/s41598-024-52220-2. 38246959 PMC10800337

