# Supplementary material for: SIRT1 Expression in Human Gastrointestinal Tumors and Its Clinical Significance
Source: Cancer Med. 2025 Sep 29;14(19):e71217. doi: 10.1002/cam4.71217 (PMC12477621; doi:10.1002/cam4.71217)
Supplement: Supplementary file 1 — Figure S1: Expression levels of SIRT1 in gastric cancer, colon cancer, and rectal cancer. Compared to normal tissue, SIRT1 expression was higher in gastric cancer patients and lower in colon and rectal cancer patients. Table S1:. Baseline characteristics of the study subject. [file CAM4-14-e71217-s001.docx]

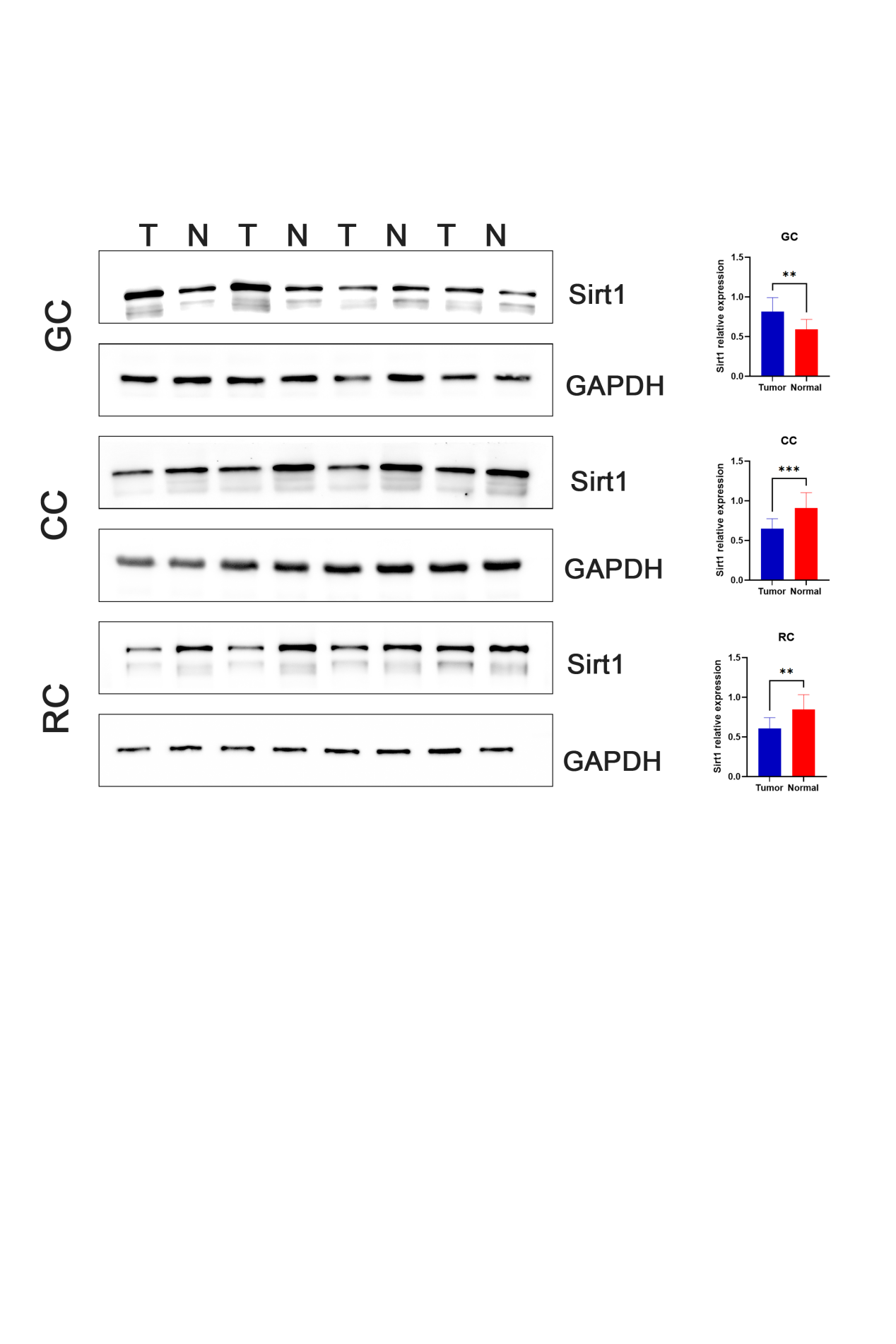


**Figure S1** Expression levels of SIRT1 in gastric cancer, colon cancer and rectal cancer. Compared to normal tissue, SIRT1 expression was higher in gastric cancer patients and lower in colon and rectal cancer patients.

| Table S1 Baseline characteristics of the study subject | | | | | |
| --- | --- | --- | --- | --- | --- |
| Parameters | Total | GC | CC | RC | control |
| Number（n） | 264 | 66 | 66 | 66 | 66 |
| Gender （n） |  |  |  |  |  |
| Male | 150 | 43 | 39 | 36 | 32 |
| Female | 114 | 23 | 27 | 30 | 34 |
| Age（years） |  |  |  |  |  |
| ‾X±S | 57.72±12.50 | 58.91±9.78 | 57.96±13.79 | 59.50±15.40 | 54.52±9.65 |
| Range | 22～83 | 34～76 | 27～80 | 22～83 | 34～74 |
| Median | 54 | 59 | 59 | 61 | 55 |
| SIRT1(ng/mL) |  |  |  |  |  |
| ‾X±S | 3.93±2.04 | 4.96±2.59 | 3.29±1.43 | 3.33±1.35 | 4.14±4.14 |
| Range | 0.15～10.19 | 0.25～10.19 | 0.15～6.35 | 0.36～5.90 | 0.28～8.71 |
| Median | 6.97 | 4.62 | 3.27 | 3.32 | 3.60 |
|  |  |  |  |  |  |
